# High DRC Levels Are Associated with Let-7b Overexpression in Women with Breast Cancer

**DOI:** 10.3390/ijms17060865

**Published:** 2016-06-02

**Authors:** Jarline Encarnación, Carmen Ortiz, Ralphdy Vergne, Wanda Vargas, Domenico Coppola, Jaime L. Matta

**Affiliations:** 1 Department of Basic Sciences, Division of Pharmacology & Toxicology, Ponce Research Institute, Ponce Health Sciences University-School of Medicine, Ponce, PR 00716-2347, Puerto Rico; jencarnacion@psm.edu (J.E.); caortiz@stu.psm.edu (C.O.); wvargas@psm.edu (W.V.); 2 Biology Department, University of Puerto Rico at Ponce, Ponce, PR 00716-9996, Puerto Rico; ralphdy.vergne@upr.edu; 3 Department of Pathology, H. Lee Moffitt Cancer Center and Research Institute, Tampa, FL 33612, USA; domenico.coppola@moffitt.org

**Keywords:** breast cancer, DNA repair, nucleotide excision repair pathway, Let-7b

## Abstract

Nucleotide Excision Repair (NER) is a critical pathway involved in breast cancer (BC). We have previously published that a low DNA repair capacity (DRC) is associated with a higher risk of BC in Puerto Rican women. Let-7b belongs to a miRNA family with tumor suppressor activity that targets oncogenes. We isolated miRNAs from plasma of 153 Puerto Rican women with and without BC. DRC was measured in lymphocytes by means of a host cell reactivation assay. These women were divided into four groups according to their DRC level: High (>3.8%) and low (<3.8%). The four groups consisted of BC patients with high (*n* = 35) and low (*n* = 43) DRC and controls with high (*n* = 39) and low (*n* = 36) DRC. Epidemiologic data were collected at initial BC diagnosis and almost five years after diagnosis. A significant difference in Let-7b expression was found in BC patients with high DRC *versus* the remaining groups (*p* < 0.001). Thus, our data reveal a possible role of Let-7b on DRC during breast carcinogenesis. Our study is innovative because it provides the first evidence that Let-7b may play role in DRC regulation (through the NER repair pathway) in BC.

## 1. Introduction

MicroRNAs (miRNAs) are endogenous, short (19–24 nucleotides) non-protein-coding RNAs that regulate gene expression at the post-transcriptional level via binding to 3′-untranslated regions of protein-coding transcripts [1,2]. miRNAs have been exhaustively studied due to their role in embryonic development [1,3]. In recent years, there has been a keen interest in how miRNAs can be used to study cancer [2,4,5,6]. They have become promising tools in identifying metastases, chemo-resistance, and cell cycle checkpoints [1,7,8]. Alterations in the expression profiles of many miRNAs have been utilized as classifiers in many cancer types [9]. Numerous studies have shown that miRNAs can be detected in serum and tissue in a stable form, showing protection from endogenous RNase activity [5,9,10]. This makes them attractive biomarkers for many cancer endpoints. Dysregulation of miRNAs have pathogenic implications in breast, colon, lung, and ovarian cancers, among others [4]. Aberrant microRNA expression is a hallmark for discriminating between normal tissue and solid tumors.

Breast cancer (BC) is the most common cancer affecting women worldwide. It accounts for 20% of all malignancies in females; in Puerto Rico (PR), it accounted for 30% of all cancers in 2012 [11]. Humans vary in their inherent sensitivities to mutagens and carcinogens due to differences in their DNA repair capacity (DRC) levels [12,13,14]. Several studies using functional repair assays in lymphocytes have demonstrated that DRC varies greatly among individuals and that lower DRC is associated with higher risk of several types of cancer [15,16,17]. Our laboratory was the first to report that a low DRC, measured in terms of the NER pathway, is an important risk factor for BC [16].

The NER pathway is the most complex DNA repair pathway and includes the repair of bulky, helix-distorting DNA lesions, such as those induced by crosslinking agents and base-damaging carcinogens and chemical mutagens [12,13,16]. Defects in this pathway are normally associated with rare genetic syndromes and cancers [18] (e.g., Xeroderma pigmentosum (XP) and Cockayne syndrome). However, Latimer *et al*. (2010) demonstrated the critical importance of the NER pathway in BC [19]. Other studies in the literature have also demonstrated that a low DRC can increase the risk of developing cancer [20,21,22,23,24,25,26,27,28,29,30,31,32]. Considered a “generalist” of DNA repair pathways, NER works in multiple capacities, particularly when other repair pathways exhibit reduced functionality. Latimer found that the mean NER capacity of the tumor samples she studied was significantly lower than that of normal breast tissues, averaging only 44% of normal activity (*p* < 0.001) [19]. Thus, learning how DRC influences one’s BC risk, disease progression/recurrence, and prognosis would be an invaluable addition to the arsenal of BC biomarkers [15,19,20]. Dysregulation of DNA repair mechanisms have been clearly linked to breast carcinogenesis and, in certain kinds of BCs, are being exploited today as potential therapeutic targets [10,16,21]. One of the epigenetic factors that may influence DRC and BC risk is microRNAs (miRNAs) [16,22]. Although there have been significant advances in our understanding of the functionality of miRNAs, their role as regulators of expression of DNA repair genes and variability in DRC is still poorly understood [22]. Studies on the role of miRNAs in DNA damage and repair have focused on miR-21, miR-24, miR-101, miR-210, and miR-155 [6,9,23]. The expression of several miRNAs within the Let-7 family has been associated with BC in previous studies [5,10]. There are no studies on the association between DRC levels and Let-7b expression in BC. Although this miRNA family has been widely studied, only a few articles have reported different types of Let-7b regulation, particularly in BC [2,4,5,9,10]. Therefore, our study brings innovation by increasing the knowledge in this field and adding the link with DRC levels. The review published by Barh (2008) predicted based on bioinformatics analysis, an upregulation of Let-7b expression in BC using the “miRGator” database [2,24].

Let-7b expression is differently regulated among BC populations and it has been lately considered as a potential biomarker in BC patients despite the discrepancies within the literature regarding its regulation [24,25]. Let-7b has been found to be involved in several mechanisms as a tumor suppressor, in order to halt cell proliferation, adhesion, and invasion by targeting genes of PKA1, DIAPH2, RDX, Ras, c-myc, and HMGA2 proteins [22,26]. Despite that, the exact mechanism underlying how these genes are suppressed is still unknown. Here we provide data that contributes to decipher some of these mechanisms and gain a better understanding of the role of DRC in Let-7b expression in BC.

We initially performed a discovery experiment using plasma from 30 BC patients and 30 controls with high and low DRC. Our results obtained with the RT-PCR TaqMan Array Human MicroRNA A Cards v 2.0 (Applied Biosystems) allowed us to select several candidate miRNAs that might be differentially expressed in BC patients and controls with high and low DRC. Let-7b was among the candidate miRNAs obtained in this discovery experiment with a *p*-value of 0.045. Therefore, the focus of this study was to compare the expression of Let-7b in women with BC with a control group using their DRC levels to determine whether such association exists. We hypothesized that if Let-7b expression is associated with DRC levels, then variability in DRC levels would alter the Let-7b transcript. To achieve this, we studied the expression of Let-7b in plasma of 73 BC patients and in 72 women without BC, with either high or low DRC levels. DRC was measured in human lymphocytes with different severities of NER deficiency by means of a host-cell reactivation phenotypic assay with a luciferase reporter gene [15].

## 2. Results

### 2.1. Sample Stratification

A Kruskal-Wallis (KW) test (nonparametric test) was performed to assess the differences among groups. The *p*-value for the KW test was <0.0001; thus a post hoc test was performed. Results from the Dunn multiple comparisons test suggested that the differences that occurred between groups were due to the high DRC levels of BC patients. No significant difference was found when the controls with high and low DRC were compared (*p* > 0.05). In addition, no significant differences were detected between the control and BC groups with low DRC (*p* > 0.05). However, significant differences were found when the control group was compared with BC patients with high DRC (*p* < 0.001). Moreover, a significant difference of Let-7b expression was detected when the high and low BC groups were compared, with higher Let-7b expression in BC patients with high DRC (*p* < 0.001).

### 2.2. Demographic Characteristics of the Control and Breast Cancer Study Groups

**Figure 1:**
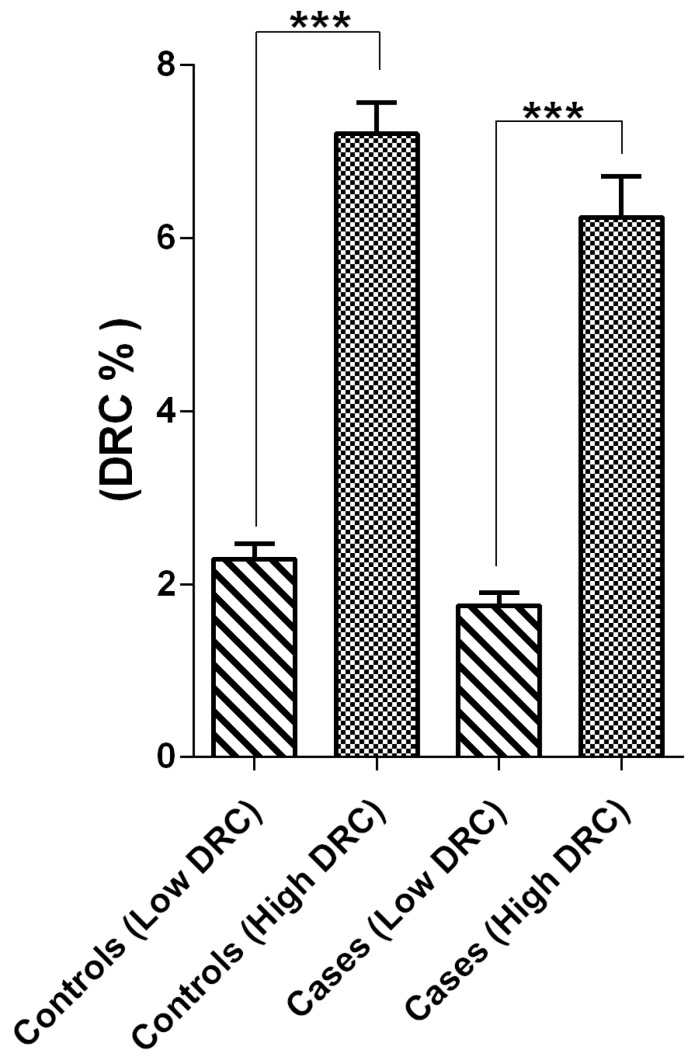
Sample stratification of 153 breast cancer patients and controls based on their DNA repair capacity (DRC) levels. These women were divided into four groups according to their DRC level: high (>3.8%) and low (<3.8%). The four groups consisted of BC patients with high (*n* = 35) and low (*n* = 43) DRC and controls with high (*n* = 39) and low (*n* = 36) DRC. Asterisk over the bars represent the statistical significance of the KW test, *** *p* < 0.001.

Our study population was composed of 153 women from a previously described cohort study [15] including women without (control) and with BC (Table 1). These groups were further classified into four subgroups according to their DRC level (Figure 1), as described in the Methods section. The demographic characteristics of the control and BC groups were evaluated using a Pearson chi-squared test. No significant association was found in the distribution between control and BC patients when divided by DRC levels (*p* = 0.4714). Thus, members of each pair of control and BC patients were matched between groups, and this cohort was used for further analysis. Most of the women in the control group were within the age range of 41–60 years, in contrast to the BC patients who were older (≥61 years) than the controls (*p* = 0.0049). In terms of body mass index, no significant association was found between the BC and control groups (*p* = 0.4045). Most of the women in both groups had reported to have been pregnant at least once (*p* = 0.2238). Despite that, a significant difference was found among BC and control women when age at first birth was compared for controls who were between 20 and 29 years old (*p* = 0.0029). No significant difference was found between BC and control groups when comparing breastfeeding practices (*p* = 0.9789). Variables, such as the use of oral contraceptives, having regular menstruation periods, age of menarche, history of endometriosis, and having a hysterectomy were not significantly different between BC patients and controls. Most of the women in our cohort did not have an oophorectomy. However, a statistical significance was found between women with BC and our control group when each group was stratified by the age at which the oophorectomy was performed (*p* = 0.0347). Women in the control group reported having an oophorectomy at a younger age compared to that of BC patients. No significant differences were observed in terms of menopause status when BC patients and controls were compared (*p* > 0.05). Controls were more likely to have received hormone replacement therapy than women in the BC group (*p* = 0.0143). When other nominal variables were analyzed to determinate whether harmful habits (smoking and alcohol consumption) contributed to differences between BC patients and controls, we found that neither contributed to these differences (*p* = 0.232 and *p* = 1, respectively). A positive family history of cancer, BC, or any other cancer did not affect our results. Most of the BC patients in our study were diagnosed with ductal (*p* = 0.0140) and invasive (*p* = 0.0019) breast carcinoma (Table 2).

### 2.3. Let-7b Expression

**Figure 2:**
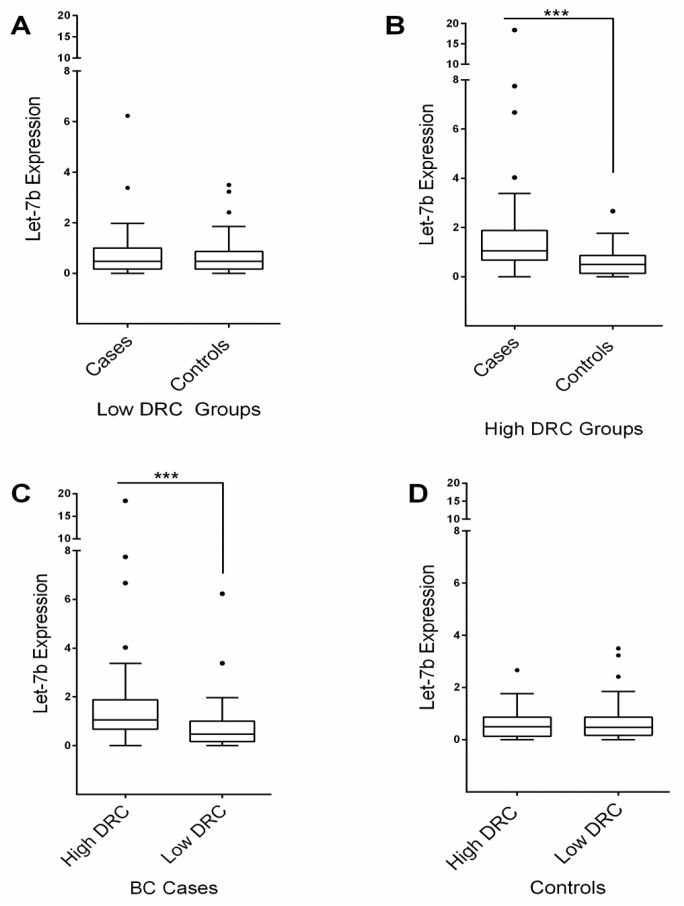
Let-7b expression measured in plasma samples of breast cancer patients and women without breast cancer (controls) with high and low DRC. (**A**) No significant differences were observed between the breast cancer patients (*n* = 43) and the control group (*n* = 37) with low DRC; (**B**) Statistically significant results were found between the breast cancer patients (*n* = 35) and controls (*n* = 39) with high DRC; (**C**) Comparison between breast cancer patients with high *versus* low DRC showed a significant difference in Let-7b expression; (**D**) No significant difference was observed when control groups with high *versus* low DRC were compared. Box plots represent the data distribution of 78 breast cancer patients and 76 controls in independent experiments. *** *p* < 0.001 using KW test.

The expression of Let-7b was analyzed based on stratification of the four experimental groups. As previously described, the groups were stratified, using a percent cut-off in low DRC (<3.8%) and high DRC (≥3.8%). The stratification by DRC resulted in two individual control groups and two individual BC groups. Control women with a low DRC (*n* = 39) had a mean expression level of 0.6 ± 0.1 (mean ± SEM). BC patients with a low DRC (*n* = 37) had a mean Let-7b expression level of 0.7 ± 0.1. The mean value for Let-7b expression for the control group with a high DRC was 0.6 ± 0.1. For the BC group with a high DRC, the Let-7b expression level was 2.0 ± 0.5 (Figure 2). The normality test was true only for the BC group with high DRC.

### 2.4. Correlations and Associations Analyses between Groups

The Spearman’s test yielded an *r* value of 0.3695, this positive fraction shows that these variables tend to increase together. The correlation between these variables was statistically significant (*p* = 0.0010) (two-tailed). Contingency tables were constructed and the Fisher’s exact test was performed to assess univariate comparisons between Let-7b expression and DRC levels. After stratifying BC patients based on their DRC levels and Let-7b expression, a significant association was found between Let-7b expression and DRC (Fisher’s exact, *p* < 0.0001).

### 2.5. Let-7b Results Based on Clinicopathological Characteristics

To determinate whether Let-7b expression was associated with clinicopathological characteristics of BC, the pathologies of the BC patients were analyzed. Women with BC having high and low DRC levels were further stratified by receptor status (estrogen receptor (ER), progesterone receptor (PR), and human epidermal growth factor receptor 2 (HER2)) and tumor grade (Table 2). These groups were also divided by molecular subtypes, including Luminal A (ER+/−, PR+/−, HER2−; *n* = 37 patients), Luminal B (ER+/−, PR+/−, HER2+; *n* = 16 patients), and triple negative (ER−, PR−, HER2−; *n* = 3 patients), conserving the division based on DRC levels. A significant difference was detected between the BC groups with a high and a low DRC based on ER-positive (*p* = 0.0007) and PR-positive (*p* = 0.0007) status alone. A significant difference was also found among the HER2-negative groups with high and low DRC levels (*p* = 0.0258). The analysis based on BC molecular subtype showed no significant differences regarding Let-7b expression among the Luminal B and triple negative BC subtypes. However, a significant association was found between Let-7b expression among the groups of Luminal A (*p* = 0.0063) with low *versus* high DRC.

## 3. Discussion

### 3.1. Let-7 Expression

miRNAs are activated by a wide variety of canonical pathways, as reported in the literature, in order to regulate different cell processes such as DNA damage response [27]. This is the first report, to the best of our knowledge that provides evidence of a possible link between Let-7b overexpression and NER pathway activity, measured with a phenotypic assay, in BC. Our results suggest that Let-7b is involved in the activity of the NER pathway, as suggested by the study of Simone *et al.*, (2009) [28]. Let-7b expression was measured in fibroblast cells after exposure to different DNA-damaging agents, including ionizing radiation, H_2_O_2_, and etoposide. After exposure to H_2_O_2_, the expression of Let-7b was induced. Among the three different DNA damaging agents, H_2_O_2_ is the only one that is known to induce the NER pathway [29,30,31]. Ionizing radiation and etoposide exposure have been shown to trigger the activation of the Base Excision Repair and DNA double-strand break repair pathways. Thus, this finding suggests and supports that Let-7b activation might be involved in the NER pathway activation. In the present study, our main objective was to examine the expression of Let-7b in BC patients *versus* a control group of women without BC using their DRC levels measured in lymphocytes as the basis for our analysis. The results validate our hypothesis that if Let-7b expression was associated with DRC levels, then variability in DRC levels would alter the Let-7b transcript. Here, we propose the possible up-regulation of Let-7b, a tumor suppressor miRNA, in BC patients with high DRC levels. Our findings reveal that a high Let-7b expression in BC patients is associated with a high DRC. Since Let-7b is a tumor suppressor miRNA, our findings support the hypothesis proposed by Giacona *et al.* (1998) and Fournie *et al.* (1995) [24,32,33]. These studies show that miRNAs are derived from the tumor necrotic tissue and have been found to enhance the expression of these non-coding molecules into the blood [10]. These miRNAs are transported through the plasma packed in endosomes. Previous studies have demonstrated that the lipids that compose these endosomes could be the result of the tumor necrotic lysis [11,34] Therefore, the miRNA expression profile is different between those with and without BC [25,35,36]. A similar trend was observed in our results where Let-7b expression was different among patients with BC and a control group. Our previous findings show that a higher activity of the NER pathway is associated with a reduced risk of BC [15]. In this study, we report that having a high DRC is associated with a high expression of Let-7b. Because the BC patients included in our study were recently diagnosed, the Let-7b expression levels measured were intrinsically from tumors in development. A possible explanation for these results could be that the high DRC levels may induce high Let-7b expression as a mechanism to reduce the cancer cell proliferation. However, more experiments are needed to test this hypothesis such as evaluating the expression level of other BC related miRNAs or the transcription level of important genes for NER pathway activity.

There are discrepancies in the literature regarding Let-7b expression in BC. In a UK study of women with BC, expression levels of Let-7b were found to be high, whereas in a study from Korea, women with BC displayed low expression levels [10,21]. In our cohort, most of the women with BC had a low DRC; therefore, it is not surprising to find more studies reporting low levels of Let-7b expression in women with BC. Our findings are pivotal and provide information that could lead to the generation of new hypotheses regarding the role of Let-7b in the circulation in combination with high DRC in BC. Future studies are needed to examine the expression of Let-7b in tumors and plasma from patients with BC to elucidate the relationship between the two. A wide spectrum of articles has highlighted the potential of miRNAs for diagnostic biomarkers development. The benefits of miRNA overexpression have been also reported in the literature due to their potential to suppress oncogenes, which makes them a possible tool for cancer therapy [14,16]. The wide implications of our findings open new avenues regarding DRC and Let7-b transcript. The control groups, with high and low DRC, showed no significant differences in Let-7b expression. These findings led us to conclude that a high expression of Let-7b in combination with high DRC levels is a response that takes place during breast carcinogenesis. Reduced Let-7b expression in the plasma of the controls allows us to suggest that this miRNA has a different function in women without BC (controls) compared with women with BC. It has been well documented that miRNAs may have a physiological role in healthy individuals [25,37,38]. However, no association has been reported regarding the levels of Let-7b expression in women without cancer [34]. It has been reported that low levels of Let-7b expression in individuals without cancer could be attributed to the process of apoptosis in lymphocytes and other nucleated cells [24]. This is a continuous process due to the presence of the DNase I and II enzymes in the circulation. These enzymes degrade the free circulating non-coding RNA and control the amounts of miRNAs that are released from the apoptotic processes [24,35]. Thus, smaller amounts of miRNAs have been found in the plasma of those without cancer.

### 3.2. Epidemiological Associations

BC and DNA damage can be caused by various factors, including aging, environmental, genetic, and epigenetic factors. Understanding the DRC as a risk factor for BC contributes to our understanding of a key mechanism associated with the development of BC. One of the intrinsic characteristics of cancer development is a deficiency in cellular DNA repair mechanisms [13,16]. Previous studies have shown that DRC levels can vary among individuals in an age-dependent manner [15,16]. However, the DRC levels in our cohort were not significantly different among the four groups despite the age differences. Most of the patients with BC in our study were 61 years of age or older. This finding correlates with previous studies from our group and others [11,15,16,39] that age is a risk factor for BC.

Most of the women in our study population reported to have been pregnant; however, a difference was observed in age at first birth. A greater number women with BC had their first child at 30 years of age or older. This finding correlates with previous studies that show that BC incidence is higher in women who had children at 30 years of age compared with those who gave birth at younger ages [40]. In terms of breastfeeding (BF), we found no significant differences between patients with BC and controls; however, this is not a typical finding since breastfeeding is considered a protective factor for BC [41]. Although oral contraceptive use is considered a risk factor associated with premenopausal BC [42,43,44], no significant differences were found between the BC and control groups. When we evaluated the history of previous surgery, such as hysterectomy and/or oophorectomy, no significant differences were found between the BC and control groups. Although most of the women in our control group reported to have had an oophorectomy before reaching 40 years of age, women with BC had it at an older age. This has been previously reported in the literature [45,46,47]. Several retrospective studies have evaluated the effects of hormone replacement therapy (HRT) in BC [40,48]. In our cohort, the BC group reported a higher use of HRT compared with the control group. This supports conclusions of previous studies that have shown that the use of HRT increases the risk of BC [40,48]. 

### 3.3. Clinicopathological Characteristics

Over the past decade, BC therapies have been based on the knowledge gained from breast tumor studies and clinical outcomes of patients exposed to different therapies [49,50,51]. These studies have led to recognition of the heterogenic diversity within BC diagnosis, and breast tumors have been classified into molecular subtypes based on hormone receptor status PR, ER, HER2 [20]. In a recent study of 270 women with BC from our cohort, we have recently reported that there is an association between DRC levels and ER status, this association is modified by HER2 receptor status [52]. Consequently, Let-7b expression was analyzed using the molecular subtypes (including ER status) from the pathology reports. Our findings show that Let-7b expression was independent on ER, PR, and HER2 status. In a previous study by Joosse *et al.* (2014) using BC cell lines representative of different molecular subtypes, including MCF-7 (luminal A), MDA-MB-231 (luminal B), and BT-474 (triple negative), among others, Let-7b was expressed in all cell lines independently of their receptor status [10]. These results support our finding in the plasma of the BC patients in our study population.

## 4. Materials and Methods

### 4.1. Patient Recruitment

Participants in this study represented a subgroup of women recruited through our larger BC study (1187 patients and controls; recruited from 2006–2013), which has been previously described [15]. All participants signed an informed consent approved by the Institutional Review Board by Ponce Health Sciences University (IRB #120207-JM). This consent provided access to patient medical records, including pathology reports. Each patient completed an epidemiologic questionnaire with the assistance of the study nurse to obtain the demographic information. All patient information was de-identified and coded and kept in a locked cabinet and in protected computers available only to those involved in conducting this study. The patients selected for this study were women with primary histopathologically confirmed diagnosis of BC and were recruited from gynecology and oncology clinics throughout Puerto Rico. Inclusion criteria were patients who (1) were recently diagnosed histopathologically with primary BC; (2) were treatment-naïve (had not received chemotherapy, blood transfusions, or radiotherapy); and (3) had pathology reports that included hormone receptor information.

### 4.2. DNA Repair Capacity Measurements

Peripheral blood samples were used to obtain lymphocytes, which were separated, purified, and grown from each patient sample. These cells, which included tumoral cells, were used as surrogate markers of the patients’ overall DRC. A host-cell reactivation assay (HCR) was performed on the peripheral blood lymphocytes to measure *in vivo* DRC, as described in previous studies [15,16]. After transfection into lymphocytes, repair-transcription-blocking damage was introduced exogenously on foreign DNA, and then overall DRC was measured via HCR. This approach measured the unaffected phenotype, which reflects the cells’ inherent DRC, measured primarily in terms of their NER activity. Keeping the time constant for lymphocytes to complete the repair mirrored the true cellular process [15].

To calculate the DRC, undamaged plasmid DNA was compared with repair of *in vitro*-damaged plasmid DNA; the results were expressed as the percentage of residual luciferase reporter gene expression (% luciferase activity in luminescence units). The amount of gene expression reflected DRC, expressed as a percentage. A detailed description of the assay, including separation of DRC by tertiles has been published [15]. This study builds on our laboratory’s 15 years of experience in performing the HCR assay to measure DRC and its validation of the sensitivity, specificity, and usefulness as a measure of BC risk.

### 4.3. Sample Stratification

We collected 156 plasma samples from women with BC and controls, from the previously described study cohort [15], which were stored at −80 °C and used to stratify the groups based on their DRC level. For this study, we used a cut-off for high *versus* low DRC levels, where a DRC higher than 3.8% was considered high and a DRC of 3.8% or less was considered low. This cut-off was established from our previous study [15] without compromising the sensitivity or specificity of the HRC assay. Study participants were further stratified into four groups: BC patients with high DRC (*n* = 32) and low DRC (*n* = 41) and women without BC (controls) with high DRC (*n* = 38) and low DRC (*n* = 34) (Figure 1).

### 4.4. mRNA Extraction and cDNA Synthesis

Total RNA from the cells lines and plasma were extracted using miRNeasy Mini Kit reagents (Qiagen, Louisville, KY, USA) [38,53]. Some modifications of the protocol were done in order to mix the RNA extraction from the plasma [54]. The total sample volume was 400 µL of plasma for all extractions. After 1 mL of QUIAZOL (Qiagen, Louisville, KY, USA) was added per sample, samples were vortexed, and 5 µL of a spike-in solution was immediately added. A concentration of 5 fmol/µL of cel-miR-39 (Ambion^®^, Austin, TX, USA) was used as spike-in. The column volume did not exceed 700 µL during the entire extraction process. The resulting extraction was used for cDNA synthesis using the Taqman^®^ MicroRNA Reverse Transcription Kit (Applied BioSystem, Frederick, MD, USA). Polymerase chain reactions (PCRs) were performed according the manufacturer’s instruction. The programmed thermal cycler for clonation was as follows: 30 min at 16 °C, 30 min at 45 °C, and 5 min at 85 °C, using a DNA Engine^®^ Thermal Cycler (PTC-200, Applied Biosystems, Foster City, CA, USA).

### 4.5. Let-7b Expression Using Real-Time PCR

**Figure 3:**
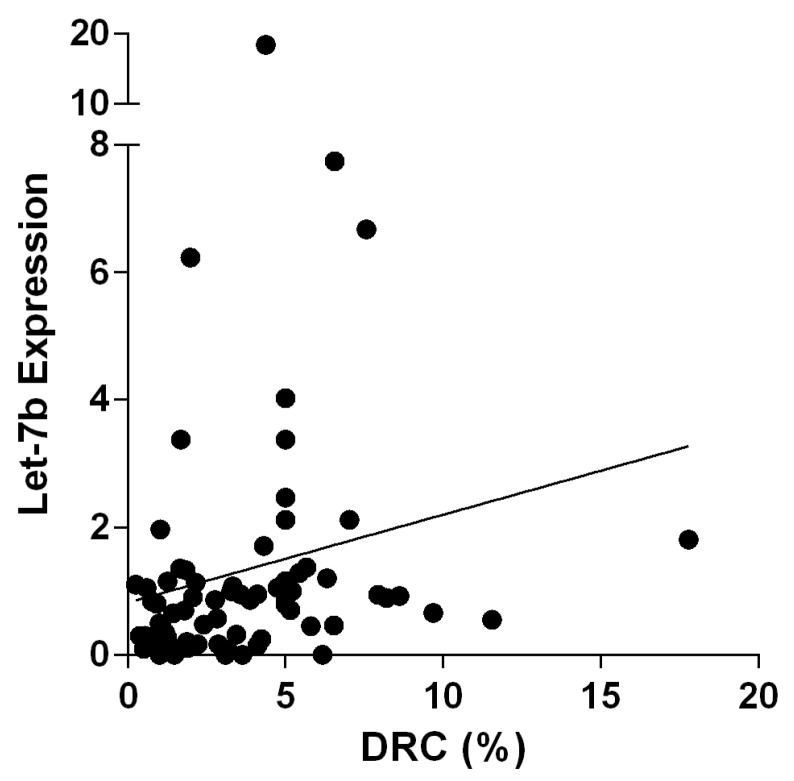
Linear regression was performed to test correlations between DNA repair capacity (DRC) levels and Let-7b expression in breast cancer patients.

Assessment of Let-7b expression was performed using TaqMan^®^ microRNA assay (Applied Biosystems, Louisville, KY, USA). A validation method was first performed as suggested by the manufacturer. Quantitative florescent amplification of cDNA of has-Let-7b, has-miR-16, and cel-miR-39 was performed using TaqMan^®^ MicroRNA Assays (Applied Biosystems, Louisville, KY, USA, USA). Real-time PCR was conducted with the use of the CFX96 TM Real-Time System (Thermal Cycler) (Bio-Rad, Hercules, CA, USA). Data analysis of PCRs was performed using the CFX Manager Program from Bio-Rad. The has-miR-16 was used to normalize the expression of the target miRNA. A mean of triplicate experiments was considered as the result for each sample. The Let-7b expression presented in Figure 2 and Figure 3 is equivalent to the resulted normalized expression ΔΔ*C*q from the PCRs. Thus, the relative quantity of Let-7b was normalized to the relative quantity of the reference gene across the samples. It has been reported that miR-16 from human plasma is the best choice to perform a suitable normalization procedure [55].

### 4.6. Statistical Analyses

To assess differences of Let-7b expression among groups, a Kruskal-Wallis test (non-parametric) was performed followed by a *post hoc* Dunn multiple comparisons test. Clinicopathological characteristics were used to assess Let-7b expression, with differences analyzed using the Kruskal-Wallis test. To determine whether epidemiological characteristics had any association with Let-7b expression, a Pearson chi-squared test was performed. Statistical significance was set at *p* < 0.05. The BC patient data was further analyzed to assess any correlations or associations between variables. The Spearman’s rank correlation test (nonparametric test) was performed to assess any dependence between the DRC and Let-7b expression (Figure 3). Contingency tables were constructed and the Fisher’s exact test was performed to assess univariate comparisons between Let-7b expression and DRC levels. Since no significant differences were found within the control groups (high and low DRC) the mean expression of Let-7b in these groups (0.6 ± 0.1) was used to establish the cutoff for high and low expression of this miRNA in BC patients.

## Figures and Tables

**Table 1:** Patient demographics of control and breast cancer patient groups.

Variable	Number of Patients (%)		*p* Value
	BC Group (*n* = 78)	Control Group (*n* = 75)	
DRC			
Low (<3.8)	43 (28)	36 (24.0)	
High (≥3.8)	35 (23.0)	39 (25)	0.4714
Age			
21–40 years	8 (10.2)	13 (17.3)	
41–60 years	34 (43.6)	46 (61.3)	0.0049
≥ 61 years	36 (46.2)	16 (21.3)	
Body mass index			
<25 kg/m^2^	24 (30.8)	30 (40.0)	
≥25 kg/m^2^	53 (67.9)	42 (56.0)	0.4045
Missing	1 (1.3)	3 (4.0)	
Ever been pregnant			
Yes	69 (88.5)	60 (80.0)	
No	9 (11.5)	15 (20.0)	0.2238
Age at first birth			
≤19 years	21 (30.4)	9 (15.0)	
20–29 years	33 (47.8)	47 (78.3)	
≥30 years	13 (18.8)	4 (6.7)	0.0029
Missing	2 (2.9)	0 (0.0)	
Ever breastfeed			
Yes	47 (68.1)	41 (68.3)	
No	22 (31.9)	19 (31.7)	<0.9789
Oral contraceptive use			
Yes	33 (42.3)	37 (49.3)	0.4779
No	45 (57.7)	38 (50.7)	
Age started oral contraceptive			
<20 years	4 (12.1)	9 (24.3)	
≥21 years	26 (78.8)	27 (73.0)	0.3811
Missing	3 (9.1)	1 (2.7)	
Regular menstrual periods			
Yes	52 (66.7)	39 (52.0)	
No	26 (33.3)	35 (46.7)	0.1118
Missing	0 (0.0)	1 (1.3)	
Age at menarche			
≤12 years	40 (51.3)	40 (53.3)	
≥13 years	37 (47.4)	35 (46.7)	0.9932
Missing	1 (1.3)	0 (0.0)	
History of endometriosis			
Yes	5 (6.4)	4 (5.3)	
No	73 (93.6)	71 (94.7)	0.7772
Hysterectomy			
Yes	19 (24.4)	17 (22.7)	
No	59 (75.6)	58 (77.3)	0.9553
Age of hysterectomy			
≤40 years	6 (31.6)	7 (41.2)	
41–49 years	10 (52.6)	10 (58.8)	
≥50 years	3 (15.8)	0 (0.0)	0.2259
Oophorectomy			
Yes	12 (15.4)	15 (20.0)	
No	66 (84.6)	59 (78.7)	
Missing	0 (0.0)	1 (1.3)	0.5255
Age of oophorectomy			
≤40 years	0 (0.0)	6 (40.0)	
41–49 years	4 (33.3)	4 (26.7)	
≥50 years	6 (50.0)	3 (20.0)	0.0347
Missing	2 (16.7)	2 (13.3)	
Menopause			
Yes	60 (76.9)	53 (70.7)	
No	18 (23.1)	22 (29.3)	0.4623
Hormone replacement therapy			
Yes	17 (21.8)	31 (41.3)	
No	61 (78.2)	44 (58.7)	0.0143
Smoking			
Yes	13 (16.7)	7 (9.3)	
No	65 (83.3)	68 (90.7)	0.232
Alcohol consumption			
Yes	15 (19.2)	14 (18.7)	
No	63 (80.8)	61 (81.3)	1
Family history of cancer (not BC)			
Yes	49 (62.8)	38 (50.7)	
No	29 (37.2)	37 (49.3)	0.4623
BC history in any family member			
Yes	23 (29.5)	14 (18.7)	
No	55 (70.5)	61 (81.3)	0.1339

To assess significant differences (*p* value) among groups, Pearson chi-squared test was performed. BC, breast cancer; DRC, DNA repair capacity.

**Table 2:** Clinicopathological characteristics of breast cancer patients.

Variable	Number of BC Patients (%)		Let-7b ^1^ *p*-Value
	Low DRC (<3.8) Group (*n* = 43)	High DRC (≥3.8) Group (*n* = 33)	
Estrogen receptor			
Positive	34 (79.1)	20 (60.6)	0.0007
Negative	4 (9.3)	7 (21.2)	*p* ˃ 0.05
Missing	5 (11.6)	6 (18.2)	
Progesterone receptor			
Positive	30 (69.8)	18 (54.5)	0.0007
Negative	8 (18.6)	9 (27.3)	*p* ˃ 0.05
Missing	5 (11.6)	6 (18.2)	
HER2			
Positive	7 (16.3)	9 (27.3)	*p* ˃ 0.05
Negative	23 (53.5)	16 (48.5)	0.0258
Missing	13 (30.2)	8 (24.2)	
Grade			
I	6 (14.0)	2 (6.1)	*p* ˃ 0.05
II	19 (44.2)	15 (45.5)	*p* ˃ 0.05
III	7 (16.3)	8 (24.2)	*p* ˃ 0.05
Missing	11 (25.6)	8 (24.2)	
Subtypes			
Luminal A	23 (53.5)	14 (42.4)	0.0063
Luminal B	7 (16.3)	9 (27.3)	*p* ˃ 0.05
Triple negative	1 (2.3)	2 (6.1)	*p* ˃ 0.05
Missing	12 (27.9)	8 (24.2)	
Site			
Ductal	39 (90.7)	28 (84.8)	0.0140
Ductal + lobular	0 (0.0)	1 (3.0)	*p* ˃ 0.05
Lobular	2 (4.7)	2 (6.1)	*p* ˃ 0.05
Missing	2 (4.7)	2 (6.1)	
Type			
*In situ*	4 (9.3)	6 (18.2)	0.0019
Invasive	38 (88.4)	27 (81.8)	*p* ˃ 0.05
Missing	1 (2.3)	0 (0.0)	

Clinicopathological characteristics of patients with breast cancer were stratified using DRC as a dichotomous variable, followed by a second stratification using the raw data of Let-7b expression. To assess to significant differences among groups, the Kruskal-Wallis test was performed; ^1^ *p*-value was based on a two-tailed test.
